# The Use of Computer-Aided Design and Computer-Aided Manufacturing (CAD/CAM) Technology for Zirconia Crowns After Laser-Assisted Crown Lengthening and Retraction: A Case Report

**DOI:** 10.7759/cureus.62021

**Published:** 2024-06-09

**Authors:** Mohan Valiathan, Ranjith Mari, Rudhra K, Angelin Fiona J, Sajid Hussain, Anitha Balaji

**Affiliations:** 1 Periodontics, Sree Balaji Dental College and Hospital, Chennai, IND

**Keywords:** cad/cam, laser, biologic width, bone loss, crown lengthening

## Abstract

Maintenance of biological width serves as a primary factor in periodontal-restorative relationships. Crown lengthening (CL) is a technique to prevent violation of biological width, with the laser method offering the advantage of surgical and patient-related outcomes. Laser CL with retraction helps with the excision of tissues, increasing the CL, maintaining the gingival contour with adequate exposure to the finish line to record the tooth preparation features. This helps to achieve the functional and esthetic outcomes essential for restorative dentistry. The marginal fit, contour, and adaptation of the crown can be further enhanced by computer-aided design and computer-aided manufacturing (CAD/CAM) technology improving patient and clinical outcomes. Hence, this case report aims to indulge the laser-assisted procedures and CAD/CAM technology to fabricate and deliver a zirconia crown maintaining the periodontal-restorative factors.

## Introduction

Crown lengthening (CL) is a procedure that involves the reshaping of the gingiva and underlying bone to increase the crown height to achieve the resistance and retention form in the prosthesis. This is done to improve the functional and esthetic outcomes in prostheses. For the restorative needs, it is indicated in the following areas: increasing clinical crown height lost due to caries, fracture, or wear; accessing subgingival caries and a perforation in the coronal third of the root; and producing a “ferrule” and space for crowns, veneers and restorations [1]. This procedure helps to avoid invading the biologic width (supracrestal connective tissue attachment) when fabricating restorations. Violation of biologic width can lead to chronic inflammation, pain, gum recession, and unpredictable bone loss [2]. Various methods of CL are apically repositioned flap with or without osseous recontouring, forced tooth eruption and gingivectomy using a scalpel, laser, or electrocautery.

Laser-assisted CL is one such technique that benefits in terms of precision, minimal bleeding, faster healing, reduced discomfort, minimal anaesthesia, and incisions. Also, gingival margin stability can be achieved, with reduced surgical time and preservation of complex gingival dimensions [3]. Diode laser ablation is effective and more reliable for CL involving soft tissue alterations [4].

Computer-aided design and computer-aided manufacturing (CAD/CAM) technology allows for the digital design and precise milling of dental restorations. The steps involved are intraoral scanning, designing restoration using software, and milling for crown fabrication [5]. Although there are various advancements in impression materials, they still exhibit inadequate precision because of impression technique, type of material, transportation, and accuracy of the impression. Patient discomfort by gag reflex or an unpleasant taste is one of the influential factors. In spite of this, the imperfect impression is difficult to alter in subsequent laboratory procedures, and this affects the marginal fit of the prostheses [6]. To optimize the fabrication of crown and prosthetic fit, digital technology plays a vital role in using scanners and software to enable precise and easy replication of the shape and contours, ensuring excellent adaptation with minimal adjustment [7]. Hence, the aim of this case report is to use CAD/CAM technology to fabricate a crown after laser-assisted CL to preserve the biologic width for functional and esthetic outcomes.

## Case presentation

**Figure 1:**
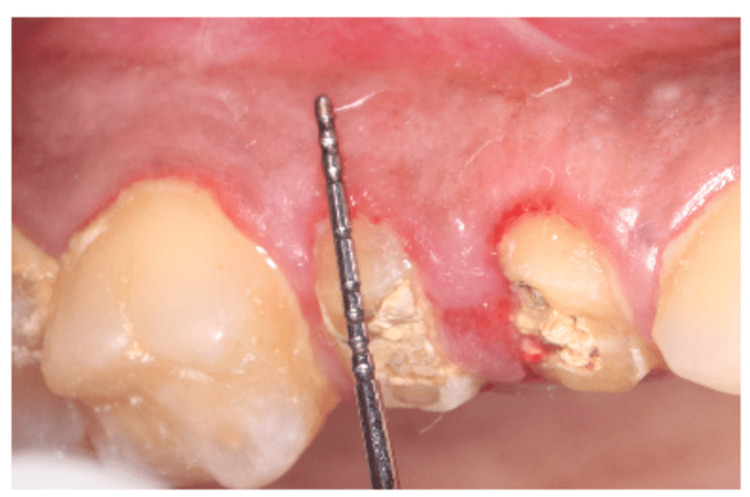
Preoperative view showing inadequate crown height in teeth 14 and 15.

A patient aged 25 years came with a complaint of reduced crown height to the department of periodontics; she had a root canal treated tooth done one month and had a shorter anatomical crown which is inadequate for dental prosthesis. On clinical examination, inadequate crown height was present in teeth 14 and 15 with 6 mm biologic width measured using bone sounding with the presence of adequate attached gingiva (Figure 1) with adequate bone height identified by periapical radiograph; hence, laser CL was planned to remove the excess soft tissue and obtain an ideal crown root ratio of 1:2 followed by single sitting zirconia crown fixation using CAD/CAM technology.

This laser-assisted CL procedure moves the marginal and interdental gingiva apically maintaining the gingival contour with the adjacent tooth. This case comes under class 2 (permanent restorations during CL procedures) of CL procedures with restorative modality given by Baghele et al. [8]. Laser retraction was also done to facilitate digital impressions and to record the finish line accurately.

Surgical procedure

**Figure 2:**
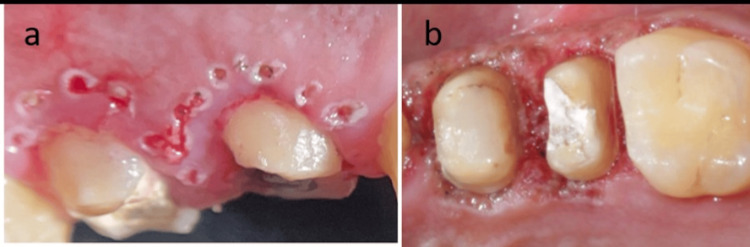
(a) Markings given using laser tip for excision; (b) the procedure involved excising 3 mm of tissue while maintaining the biologic width.

After tooth preparation with a deep chamfer line in teeth 14 and 15, a laser gingivectomy was performed (Figure 2a). Under local anaesthesia (LA), the diode laser unit was activated at 1.5 watts in continuous wave (CW) mode. The excision of tissue was done by back-and-forth brush-like strokes with gradual progression. The laser tip was used with constant movement during the procedure and the gingival margin contour was maintained by removing excess ablated tissues (Figure 2b).

**Figure 3:**
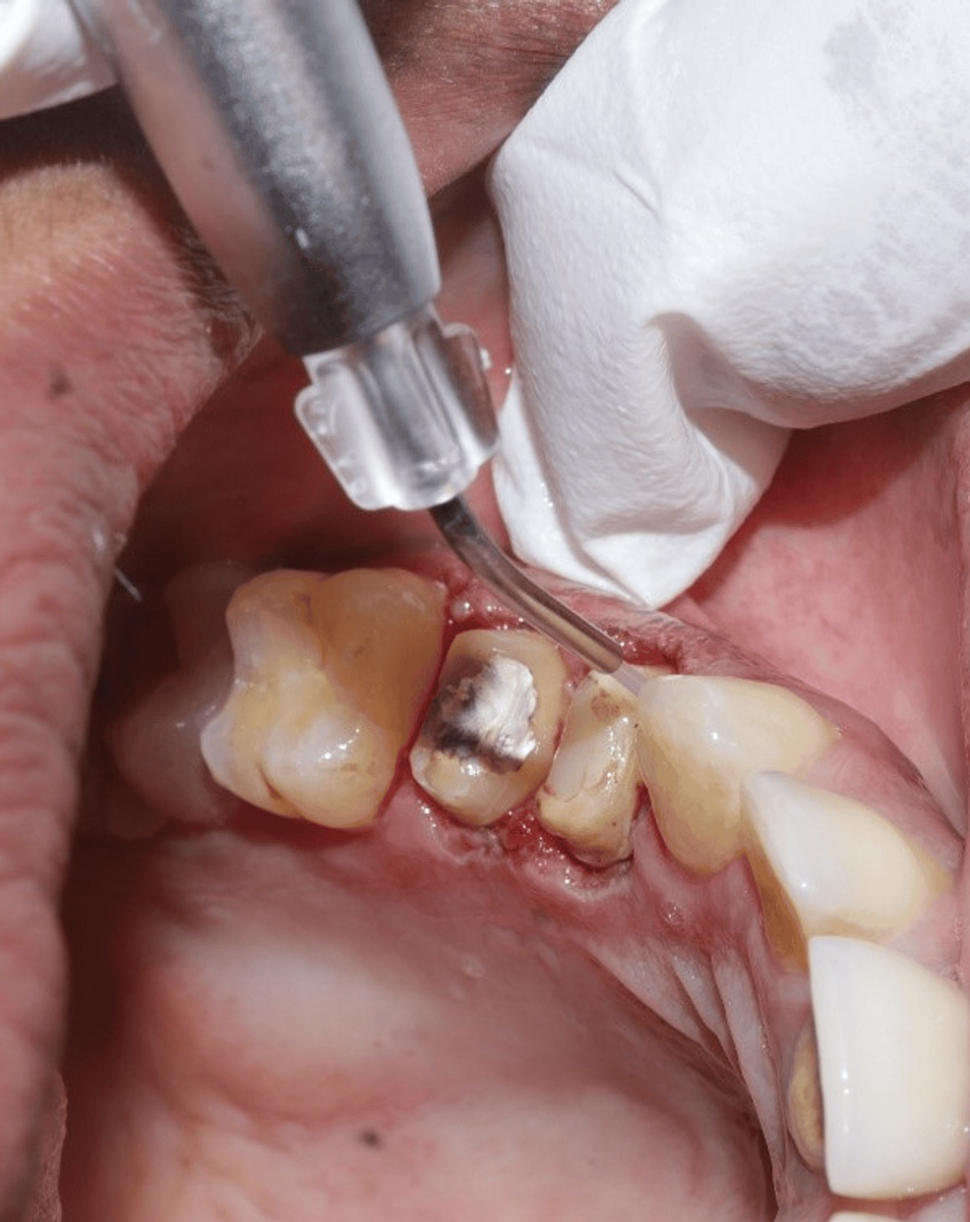
Gingival retraction done by removing the sulcular epithelium to expose the finish line.

The distance between the alveolar crest and the gingival margin was maintained at 3 mm as given by Ingber et al. (1977) to preserve the biologic width which is measured using bone sounding. Also, the laser retraction procedure was done by passing fibre optic in contact mode along the gingival sulcus, to remove sulcular epithelium at 0.8 W. A laser tip was inserted into the gingival sulcus, to record the finish line (Figure 3).

**Figure 4:**
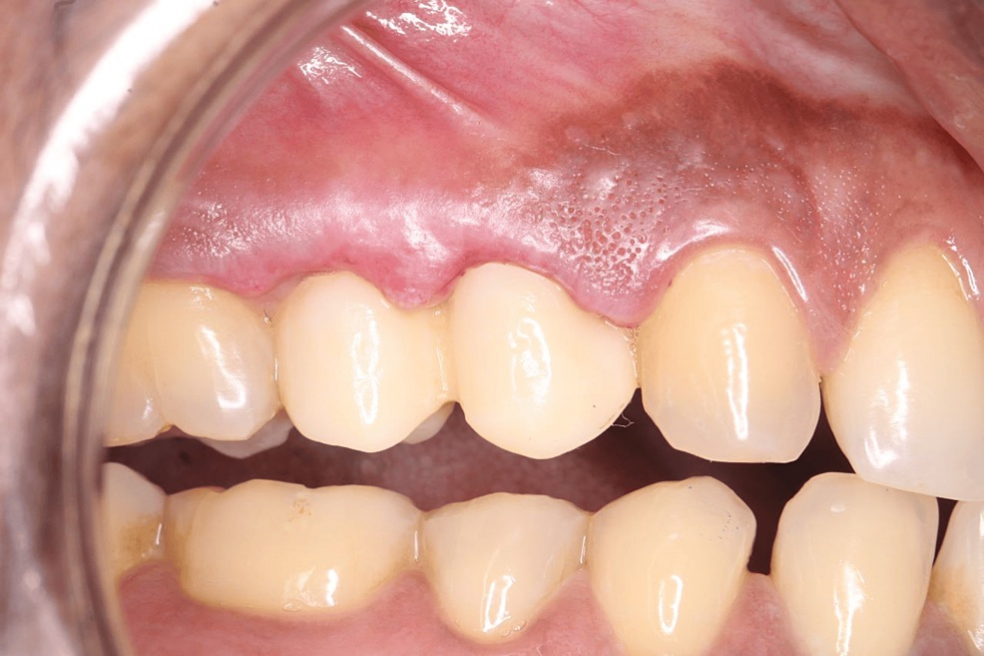
The zirconia crown was fabricated using CAD/CAM technology and then delivered. CAD/CAM: computer-aided design and computer-aided manufacturing

CAD/CAM crown fabrication was performed as follows: intraoral scanning was done to facilitate digital impression using a CEREC intraoral scanner (Dentsply Sirona, York, USA), and the crown superstructure was planned using Bluesky software (Dentsply Sirona, York, USA). Subsequently, the crown was milled and processed using a Dentsply Sirona milling machine with zirconia monoblock. The zirconia crown was delivered for teeth 14 and 15 (Figure 4). Post-operative instructions were given. No pain or discomfort was experienced by the patient. The patient was satisfied with the fit of the crown. Postoperative review after four weeks, three months and six months were satisfactory.

## Discussion

Increasing the clinical CL is essential for obtaining proper functional prosthetic outcomes. The major factor determining the crown superstructure's success is the marginal fit to prevent cement dissolution. Laser-assisted CL favours tissue excision with minimal surgical discomfort and relapse of soft tissue followed by laser retraction and enhances accurate recording of tooth preparation features and finish line using CAD/CAM technology [8].

To ensure adequate form, function, esthetics, and comfort of the prosthetics and dentition, an adequate understanding of periodontal and restorative relation is essential which depends on the concept of biologic width [9]. Ingber et al. suggested 3 mm of adequate “biologic width” which is composed of 1 mm supracrestal connective tissue, 1 mm junctional epithelium, and 1 mm for sulcus depth on an average even when margins are placed subgingivally [10].

Violation of the biologic width is a common practice in restorative dentistry with subgingival restoration. Maintenance of ideal crown root ratio and ferrule effect is essential in perio-restorative relation [11]. CL is a procedure to prevent violation of biologic width, in this laser-assisted CL presents with minimal tissue placement less tissue rebound, and better gingival stability than traditional CL [12]. Laser gingivectomy benefits with faster healing, reduced pain and bleeding with minimal post-operative complications [13]. Diode lasers act as a substitute for conventional retraction methods to facilitate visualization of preparation margins for an accurate impression [14]. Further fabrications of crowns with CAD/CAM technology enhance precise scanning techniques for accurate contour replication with minimal adjustments. It also benefits ease of communication with the storage of digital data for further modifications [15]. This zirconia crown fabricated by this technique has provided adequate marginal fit, contour, and adaptation with functional and esthetic outcomes.

## Conclusions

This case report demonstrates the successful application of laser-assisted CL combined with CAD/CAM technology for the fabrication of zirconia crowns. The integration of these advanced techniques ensured the preservation of biologic width, optimal crown-to-root ratio, and improved functional and esthetic outcomes. Laser-assisted CL allowed for precise tissue excision and minimal surgical discomfort, while laser retraction facilitated accurate recording of tooth preparation features. The CAD/CAM technology provided precise and efficient crown fabrication, resulting in an excellent marginal fit, contour, and adaptation with minimal adjustments needed. This approach highlights the importance of understanding periodontal-restorative relationships and showcases the advantages of modern dental technologies in achieving high-quality prosthetic restorations.
